# Implications of the Digestion of Milk-Based Formulations for the Solubilization of Lopinavir/Ritonavir in a Combination Therapy

**DOI:** 10.1021/acs.molpharmaceut.3c00072

**Published:** 2023-03-15

**Authors:** Malinda Salim, Gisela Ramirez, Andrew J. Clulow, Adrian Hawley, Ben J. Boyd

**Affiliations:** † Drug Delivery, Disposition and Dynamics, Monash Institute of Pharmaceutical Sciences, Monash University (Parkville Campus), 381 Royal Parade, Parkville, Victoria 3052, Australia; ‡ Australian Synchrotron, ANSTO, 800 Blackburn Road, Clayton, Victoria 3168, Australia; § Department of Pharmacy, University of Copenhagen, Universitetsparken 2, Copenhagen 2100, Denmark

**Keywords:** milk, infant formula, lopinavir, ritonavir, digestion, drug solubilization, X-ray scattering, combination therapy

## Abstract

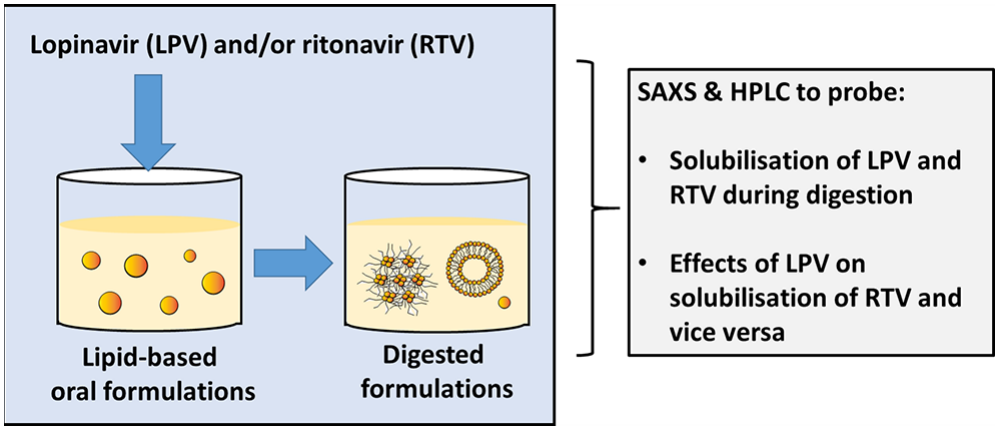

The development of formulation approaches to coadminister lopinavir and ritonavir antiretroviral drugs to children is necessary to ensure optimal treatment of human immunodeficiency virus (HIV) infection. It was previously shown that milk-based lipid formulations show promise as vehicles to deliver antimalarial drugs by enhancing their solubilization during the digestion of the milk lipids under intestinal conditions. In this study, we investigate the role of digestion of milk and infant formula on the solubilization behavior of lopinavir and ritonavir to understand the fate of drugs in the gastrointestinal (GI) tract after oral administration. Small angle X-ray scattering (SAXS) was used to probe the presence of crystalline drugs in suspension during digestion. In particular, the impact of one drug on the solubilization of the other was elucidated to reveal potential drug–drug interactions in a drug combination therapy. Our results showed that lopinavir and ritonavir affected the solubilization of each other during digestion in lipid-based formulations. While addition of ritonavir to lopinavir improved the overall solubilization of lopinavir, the impact of lopinavir was to reduce ritonavir solubilization as digestion progressed. These findings highlight the importance of assessing the solubilization of individual drugs in a combined matrix in order to dictate the state of drugs available for subsequent absorption and metabolism. Enhancement in the solubilization of lopinavir and ritonavir in a drug combination setting *in vitro* also supported the potential for food effects on drug exposure.

## 1 Introduction

Milk provides an important source of energy and nutrients necessary for the growth and development of children. Consumption of milk in the early stages of life is ubiquitous and, as such, has often been used as a vehicle for drug administration. Assessing the performance of drugs following intake of milk (and other similar foods) is therefore recommended in the development of pediatric drug formulations, as was recently outlined by the Food and Drug Administration (FDA).^1^ However, *in vitro* techniques to study the effect of milk on drug behavior during digestion are limited.^2^

Milk has also been explored as a formulation excipient for poorly water soluble lipophilic drugs since it is a natural lipid-based formulation,^3−7^ which can facilitate drug solubilization during digestion in the gastrointestinal tract to enhance oral bioavailability.^8−10^ Our group has previously shown that solubilization of poorly water soluble drugs such as artefenomel (OZ439),^9^ ferroquine,^10^ and clofazimine^11^ in the small intestinal condition during digestion was improved when coadministered with full fat milk, and similar behavior has also been observed *in vivo*.^12,13^ The enhanced solubilization of these drugs was primarily attributed to the release of fatty acids during the digestion of triglycerides in milk by the gastrointestinal enzymes.^9,10^ However, given the inherent variability of milk composition, translating milk as a viable lipid-based formulation in clinical settings is challenging. Hence, consideration of alternative milk-like systems including infant formula is an important development in this area.

Recent studies have explored the use of infant formulas as potential milk substitutes to deliver poorly water soluble drugs by investigating key lipid components that dictate drug solubilization in milk and infant formulas during digestion.^14^ It was found that the extent to which drugs could be solubilized in milk and infant formulas during digestion was greatly dependent on the amount and types of fatty acids constituting the triglycerides and on the presence of a partner drug in a combination drug therapy where two or more drugs were coadministered. For example, in a fixed dose combination of antimalarial drugs artefenomel/ferroquine, the solubilization and oral bioavailability of artefenomel was found to be reduced when ferroquine was administered concurrently.^14^ This highlighted the importance of understanding the fate of drugs when combined in a lipid-based formulation.

Lopinavir (also known as ABT-378)^15^ and ritonavir (ABT-538)^16^ are protease inhibitors that are used in a combination drug therapy to treat HIV (human immunodeficiency virus) infection in adults and children. The pharmacokinetics of lopinavir and ritonavir have been widely reported,^15,17−19^ and it has been widely accepted that ritonavir is a “pharmacokinetic booster” for lopinavir, i.e., coadministration of ritonavir with lopinavir improved the oral bioavailability of the latter by inhibiting the cytochrome P450 3A (CYP3A) enzymes in the intestine and liver, thereby reducing the first-pass metabolism of lopinavir.^15,20^ However, *in vitro* studies designed to indicate any contribution of mutual enhancement or inhibition of solubilization of lopinavir and ritonavir in the GI tract to changes in absorption and bioavailability are still lacking. In the context of milk and infant formula as potential vehicles for these drugs in low economy settings, this question is important for this combination of drugs and also for future combination therapies more generally.

**Figure 1:**
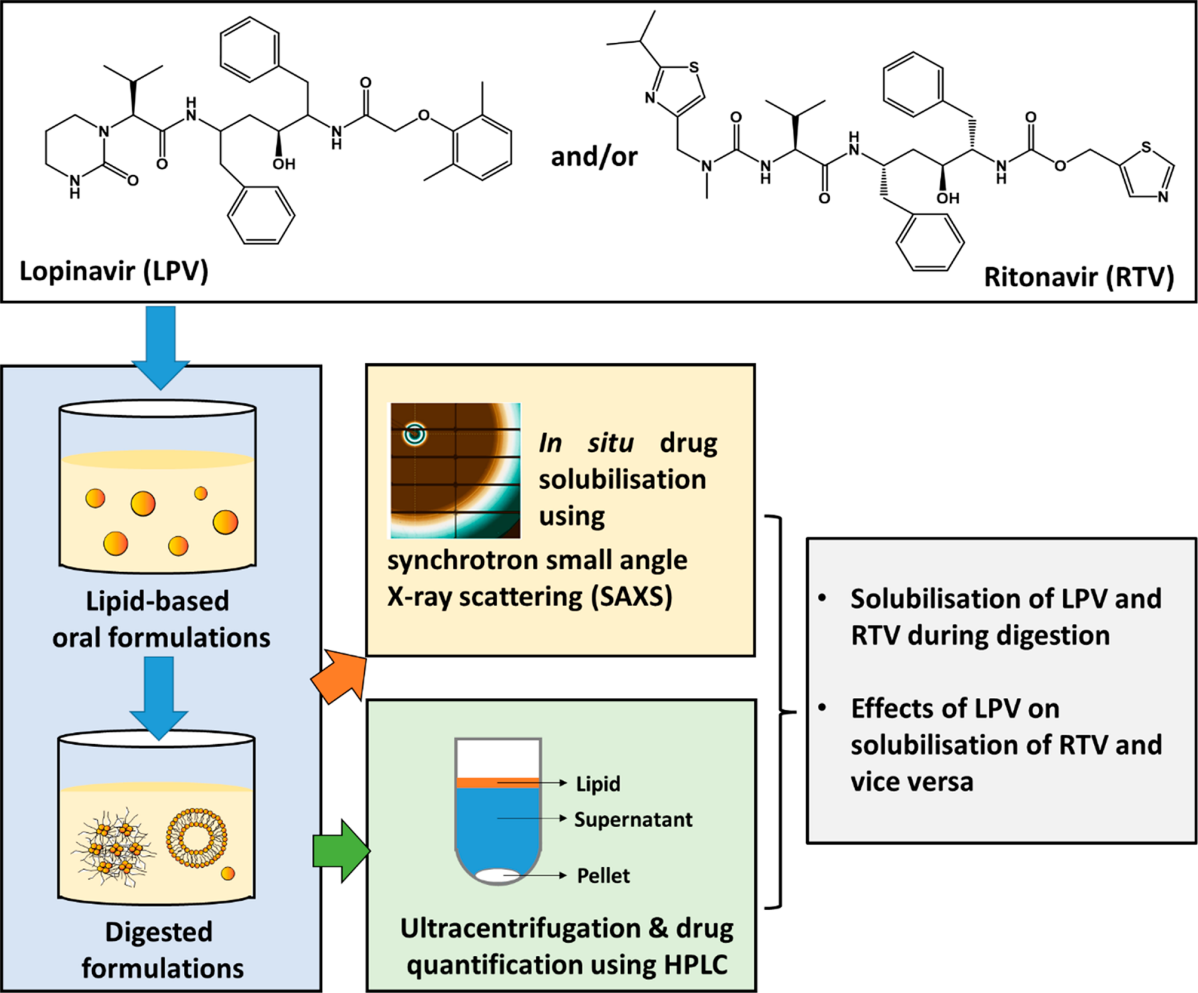
Schematic representation of the concept of this study. The impact of lopinavir and ritonavir on their respective solubilization during digestion in combination in milk and infant formula was assessed using small-angle X-ray scattering and HPLC.

Therefore, in this study, the solubilization behavior of lopinavir and ritonavir in milk-based formulations undergoing *in vitro* digestion was investigated with the aim to (1) understand how the digestion of milk and infant formula can affect drug solubilization and (2) elucidate the impact of ritonavir on solubilization of lopinavir and vice versa at different dose ratios. Figure 1 shows a schematic representation of the concept of study. These studies were performed using the drug substance/active pharmaceutical forms of lopinavir and ritonavir rather than formulated drug product to eliminate factors relating to pharmaceutical excipients in the commercially available lopinavir/ritonavir formulations. To elucidate the impact of triglyceride in non-milk components on drug solubilization, the solubilization behavior of lopinavir and ritonavir in formulated triglyceride emulsions during digestion was also investigated. Synchrotron small-angle X-ray scattering (SAXS) was used to characterize the solid-state forms of lopinavir and ritonavir and to monitor changes in concentration of crystalline drug forms *in situ* during *in vitro* digestion under simulated small intestinal conditions. The concentration of drugs partitioned into the digestion phases of the lipid-based formulations were separated by ultracentrifugation, *viz*. solid precipitate, aqueous phase containing different colloidal structures, and residual lipid phase, were measured using high performance liquid chromatography (HPLC) to quantify the amount of drugs dissolved in each phase.

## 2 Materials and Methods

### 2.1 Materials

Lopinavir (>99%) and ritonavir (>99%) were purchased from Haihang Industry Co. Ltd. (Shandong, China); and the drugs were micronized with mortar and pestle prior to experiments. Bovine milk (Paul’s brand, 3.8 w/v% fat) was purchased from a local supermarket (Victoria, Australia). Infant formula (brand not disclosed due to commercial confidence) was provided by Medicines for Malaria Venture (MMV). Nutritional information and fatty acid compositions of the infant formula have been previously reported (IF2 in the reference).^14^ Medium chain triglycerides containing caprylic/capric acids (MCT; Labrafac WL 1349) were a gift from Gattefossé (Saint-Priest, France). Long chain triglycerides rich in *sn*-2 palmitate (LCT; Infat CC) were a gift from Enzymotec Ltd., (Migdal Haemek, Israel). Milk fat globular membrane (MFGM; a whey protein concentrate containing milk phospholipids, Lacprodan MFGM-10) was kindly provided by Arla Foods Ingredients Group P/S (Viby J, Denmark). Trizma maleate reagent grade, 4-bromophenylboronic acid (4-BPBA; ≥ 95%), ammonium acetate for HPLC, sodium taurodeoxycholate hydrate (NaTDC, ≥ 95%), and sodium azide (>99%) were purchased from Sigma-Aldrich (St. Louis, Missouri). DOPC (1,2-dioleoyl-*sn*-glycero-3-phosphocholine) was purchased from Cayman Chemical (Michigan, USA). Sodium chloride (>99%) was purchased from Chem Supply (South Australia, Australia). Calcium chloride dihydrate (>99%) and sodium hydroxide pellets (min. 97%) were purchased from Ajax Finechem (New South Wales, Australia). Hydrochloric acid (36%) was purchased from LabServ (GNA Analytical Ltd., Longford, Ireland). Lipase (USP grade pancreatic extract) was purchased from Southern Biologicals (Victoria, Australia). Methanol HPLC grade (LiChrosolv) and dichloromethane (DCM) were purchased from Merck. Water was deionized and distilled (18 MΩ cm^–1^, 25 °C) using a Milli-Q water purification system (Merck, New Jersey, USA). All the chemicals were used without further purification.

### 2.2 Preparation of Drug/Lipid Formulations and In Vitro Digestion Experiments

Lopinavir (50 mg), ritonavir (50 mg), or mixtures of lopinavir:ritonavir at specified ratios (Table 1) were added to 2.75 mL of water containing 0.25 mL of 1 M HCl and vortex mixed for about 5 min to mimic a gastric condition. The acidified drug suspension was added to 17.5 mL of 3.8 w/v% fat milk or infant formula prepared at the same fat content in a digestion buffer. The drug:lipid ratios are presented in Table 1. The digestion buffer was a tris buffer at pH 6.5 containing 50 mM Trizma maleate, 5 mM sodium chloride dihydrate, 150 mM sodium chloride, and 6 mM sodium azide. Digestion was initiated by injection of 2.25 mL of lipase (about 700 tributyrin unit/mL digest) after pH adjustment of the drug/milk or infant formula samples to 6.500 ± 0.003. The pH of the samples during digestion was maintained at 6.5 by automated dosing of 2.0 M NaOH using a pH-STAT controller (Metrohm 902 STAT titration system).

**Table 1:** Ratios of Lopinavir:Ritonavir Tested, Corresponding Amounts of Drugs Added into the Lipid Formulations, and Drug to Lipid Ratios in mg of Drug/g of Lipid

Lopinavir: ritonavir ratio	Lopinavir (mg)	Ritonavir (mg)	Lopinavir:lipid ratio (mg/g)	Ritonavir:lipid ratio (mg/g)
1:1	50	50	75.2	75.2
1:4	25	100	37.6	150.4
4:1	100	25	150.4	37.6

Suspensions of lopinavir and ritonavir in simple triglyceride emulsions (3.8 w/v% fat MCT and LCT) were prepared in tris buffer in the presence or absence of bile salt micelles (4.7 mM NaTDC and 0.98 mM DOPC).^21^ Lipids from the MCT or LCT (0.76 g) were mixed with 0.11 g of MFGM in 10 mL of tris buffer (or buffer containing bile salt micelles), and the mixtures were sonicated at 25 amplitude, 2 s on/off for 3 min (for MCT) or 4 min (for LCT) using a Misonix S-4000 ultrasonic processor (New York, USA). Samples for LCT were incubated in a 60 °C oven for 5–10 min prior to sonication to melt the lipids. The resulting volume of the dispersed MCT and LCT after sonication was adjusted to 20 mL using tris buffer (or a buffer containing bile salt micelles). The MCT or LCT emulsions (17.5 mL) were subsequently added to the acidified lopinavir and/or ritonavir suspensions, and the drug/lipid mixtures were digested using 2.25 mL of lipase. The pH of the samples during digestion was maintained at 6.5 using either 2.0 M NaOH (for MCT) or 0.2 M NaOH (for LCT).

### 2.3 Synchrotron SAXS Measurements

#### 2.3.1 Static Capillary Measurements

Lopinavir and ritonavir drug powders were loaded into glass capillaries (1.5 mm outer diameter and 80 mm length; Charles Supper company, MA, USA). The capillaries were mounted in the X-ray beam of the SAXS/WAXS beamline at the Australian Synchrotron (ANSTO) (energy of X-rays selected = 13.0 keV equivalent to wavelength = 0.954 Å), and scattering patterns of the powders were acquired with a 1 s acquisition time using a Pilatus 2 M detector. The sample-to-detector distance was about 560 mm to cover a *q* range between 0.04 and 1.96 Å^–1^, where *Q* is the scattering vector defined as (4π/λ)sin(2θ/2), 2θ is the scattering angle, and λ is the X-ray wavelength. The 2D SAXS images recorded were reduced to functions of *I*(*q*) versus *q* by radial integration using the in-house software ScatterBrain (version 2.71). X-ray scattering patterns of lopinavir and ritonavir powders after dispersion in 0.1 M HCl solution were also obtained to observe any solid-state changes occurring under gastric conditions: the powdered drugs were incubated for 3 h in 0.1 M aqueous HCl and filtered using a nylon membrane (0.45 μm, 47 mm; Merck Millipore, MA, USA) and the solids were dried overnight in a vacuum oven at room temperature before being transferred to a capillary for scattering measurements.

#### 2.3.2 Flow-through Measurements

The pH-STAT apparatus used for *in vitro* digestion of the drug/lipid formulations (details in section 2.2) was interfaced with the SAXS/WAXS beamline. The digesting sample from the digestion vessel was aspirated using a peristaltic pump at approximately 10 mL/min to a fixed quartz capillary mounted in the X-ray beam (photon energy = 13.0 keV, wavelength = 0.954 Å) with a sample-to-detector distance of about 560 mm that covered a *q* range of 0.04 < *q* < 1.96 Å^–1^. 2D SAXS images were recorded using the Pilatus 2 M detector with 5 s acquisition time and 15 s delay between measurements (one measurement every 20 s); and the raw data was reduced to *I*(*q*) versus *q* by radial integration using the in-house software ScatterBrain version 2.71. The areas of characteristic diffraction peaks from each drug in the *I*(*q*) versus *q* plots were integrated using Origin software, version 2020b.

### 2.4 Partitioning of Digested Phases and Quantification of Drugs Using HPLC

To quantify the amount of lopinavir and ritonavir partitioned into digested phases of the lipid-based formulations, the *in vitro* digestion of milk, infant formula, and simple triglyceride emulsions containing the drugs was performed using the methods described in section 2.2. After 60 min of digestion, samples (200 μL) were collected into ultracentrifuge tubes and mixed with 2 μL of 0.5 M 4-BPBA (prepared in methanol) to inhibit the enzymatic activity of the pancreatic lipase. The samples were ultracentrifuged at 434 900 g for 40 min at 37 °C (Optima MAX-TL ultracentrifuge, Beckman Coulter, IN, USA) and the resultant layers (that typically consisted of an upper lipid layer, an aqueous supernatant layer, and a bottom pellet layer) were collected separately. Lopinavir/ritonavir was extracted from the individual layers using mixtures of methanol/DCM (1:1 volume ratio) and diluted with mobile phase (65% buffer B: 35% buffer A, v/v) prior to drug separation and quantification using HPLC equipped with a UV detector (Shimadzu Nexera X2, Shimadzu Corporation, Japan). Buffer A was 10 mM ammonium acetate in water, pH 4.8 and buffer B was methanol. Separation of lopinavir and ritonavir was performed using a C18 column (Waters Symmetry, 4.6 mm ID, 75 mm length, 3.5 μm particle size, 100 Å pore size) at 35 °C on a binary gradient: 65–85 v/v% buffer B for 8 min, 85 v/v% buffer B for 1 min, and 65 v/v% buffer B for 3.5 min. The injection volume was 20 μL, the flow rate was 1 mL/min, and lopinavir and ritonavir were detected at 210 nm with retention times of 6.0 and 4.9 min, respectively.

## 3 Results

### 3.1 Solid-State Forms of Lopinavir and Ritonavir

**Figure 2:**
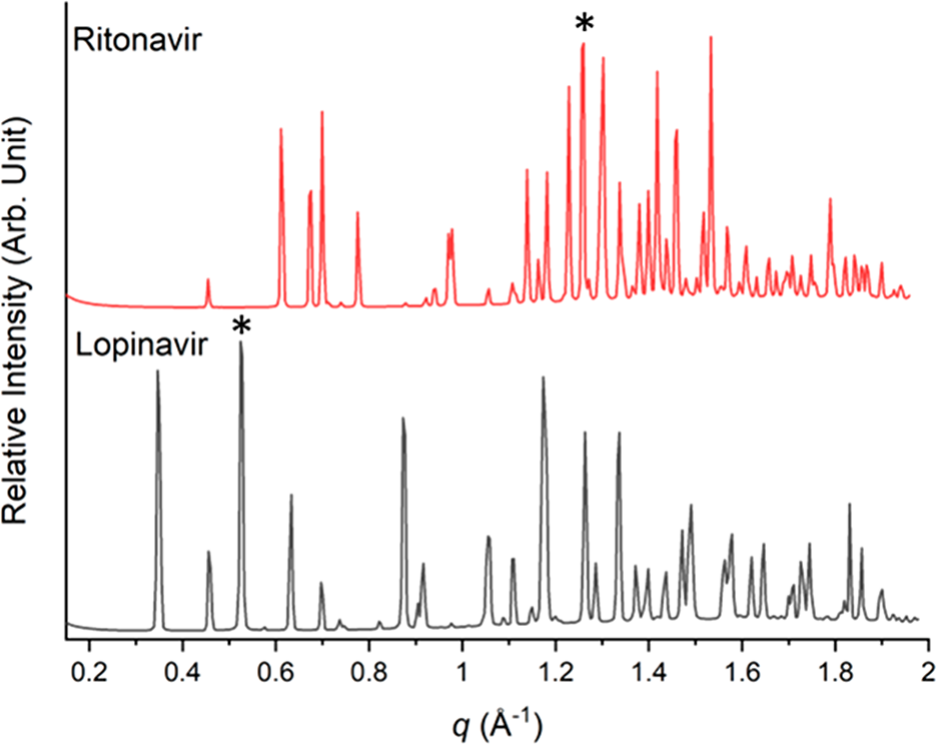
X-ray scattering patterns of lopinavir and ritonavir powders, showing the presence of crystalline drugs. The peaks with asterisks denote those used for integration in in situ solubilization experiments.

As with many small molecule drugs, lopinavir and ritonavir are known to exist as different polymorphic/pseudopolymorphic forms,^22−24^ which can potentially affect their dissolution behavior and oral bioavailability. Identification of the solid-state forms of lopinavir and ritonavir and changes that can potentially occur following exposure to gastrointestinal conditions is therefore imperative for oral formulation development. Figure 2 shows the X-ray scattering patterns of the commercially obtained lopinavir and ritonavir drug powders (no excipients), which are characteristic of a form II polymorph for ritonavir^22,23^ and a type III desolvated crystal form for lopinavir.^24^ Microscopy images of the nonmicronized drug crystals are shown in Figure S1. The diffraction peak positions for lopinavir and ritonavir (summarized in Table S1) remained consistent during dispersion and digestion in milk, infant formula, and triglyceride-based formulations, suggesting no solid-state changes occurred. Similarly, dispersions of lopinavir and ritonavir powders in 0.1 M HCl aqueous solution representing gastric pH also did not lead to polymorphic transformations (Figure S2).

### 3.2 Effects of Ritonavir on the Solubilization of Lopinavir in Lipid-Based Formulations during Digestion

Synchrotron small-angle X-ray scattering was used to monitor the solubilization behavior of lopinavir during digestion of lipid-based formulations by tracking the disappearance of the characteristic Bragg peaks shown in Figure 2. The decrease in peak area is attributed to the loss of crystallinity through dissolution or amorphization, whereas an increase in peak area is related to drug crystallization or precipitation. The appearance of new peaks indicates the formation of a new polymorphic form of crystalline drug.

**Figure 3:**
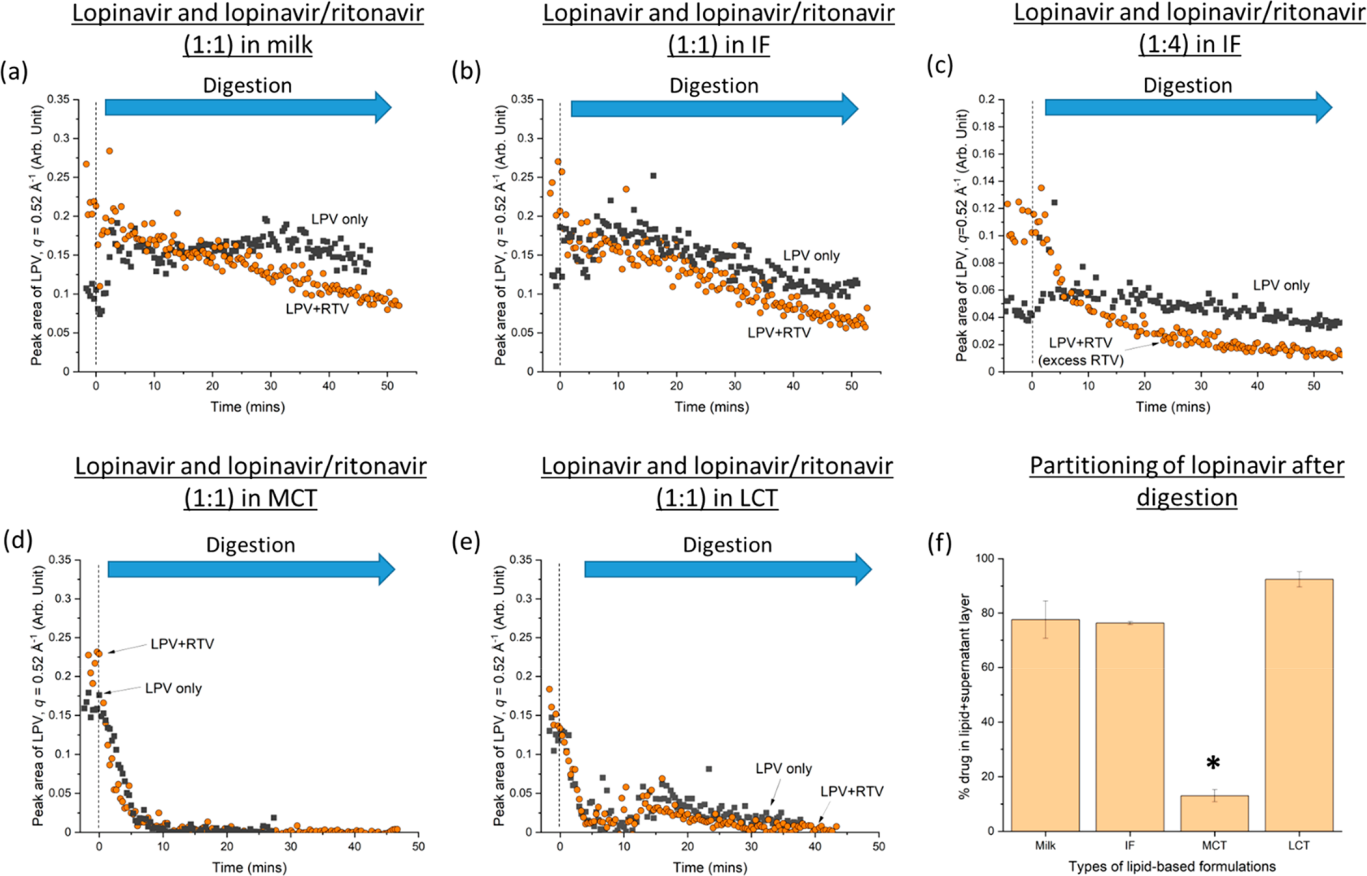
Residual crystalline lopinavir determined by area under the diffraction peak of lopinavir at *q* = 0.52 Å^–1^ remaining during dispersion (time < 0 min) and digestion (time > 0 min) of lopinavir (LPV only) and lopinavir+ritonavir (LPV+RTV) in (a) milk (LPV:RTV = 1:1), (b) infant formula (IF; LPV:RTV = 1:1), (c) infant formula (LPV:RTV = 1:4), (d) medium chain triglycerides (MCT; LPV:RTV = 1:1), and (e) long chain triglycerides (LCT; LPV:RTV = 1:1). (f) Partitioning of lopinavir in the lipid+supernatant layers (or supernatant only for MCT) of the digested lipid-based formulations at 60 min. Asterisk in panel f points to the artefactually low percentage of drug solubilized in MCT, as it was not possible to discriminate between the precipitated drug and drug solubilized in the lipid digestion phases.

Changes in the peak area attributable to lopinavir at *q* = 0.52 Å^–1^ (the peak with the strongest intensity) during the digestion of milk and infant formula are shown in Figure 3a and b. Only a very slight decrease in the area under the peak for lopinavir was observed during the digestion of infant formula, and no observable decrease was seen for milk, indicating little or no drug solubilization during the digestion of milk or infant formula had occurred when lopinavir was present alone. Addition of ritonavir to lopinavir at 1:1 weight ratio also resulted in a slight decrease in the peak area over time, possibly slightly greater than for lopinavir alone, but the effect of digestion on drug solubilization was not as substantial (Figure 3a and b) compared to when excess ritonavir (1:4 lopinavir:ritonavir weight ratio) was added to lopinavir (Figure 3c). Interestingly, when excess ritonavir (1:4 lopinavir:ritonavir weight ratio) was added to lopinavir, the initial solubility of lopinavir was reduced, evident from the greater lopinavir peak area prior to digestion (time <0 min); however, digestion resulted in improved solubilization of lopinavir, with almost all of the lopinavir solubilized by the lipid digestion products at 60 min digestion (Figure 3c).

Milk and infant formula contain a complex mixture of triglycerides, and to gain further insight into the role of fatty acid chain length on solubilization of lopinavir, solubilization during digestion was investigated using formulated emulsions prepared using medium chain triglycerides and long chain triglycerides. Figure 3d, e compares the solubilization behavior of lopinavir in the presence and absence of ritonavir during digestion of emulsified MCT and LCT under the same digestion conditions as milk and infant formula. In both cases, a rapid and complete disappearance of the peak attributable to lopinavir was observed after initiation of digestion. Lopinavir was solubilized particularly rapidly by the digesting MCT formulations; additional experiments at lower fat content demonstrated complete peak disappearance when using a fat content as low as 1 w/v%, which is equivalent to 285.7 mg of drug/g of lipid (Figure S3).

Typically, when centrifuging emulsions after digestion, a lipid layer and an aqueous supernatant layer may exist from which drug is hypothesized to be available for absorption, and a pellet layer containing solid precipitated drug may also be present, which is assumed to represent the unavailable fraction, with the goal of formulation being to maximize the amount of drug in the lipid+supernatant layers. Quantification of lopinavir in the different layers using HPLC showed in the case of the digested LCT emulsion that a higher amount of drug was present in the lipid+supernatant layer compared to milk and infant formula (Figure 3f). These observations were consistent with the observations from SAXS that a less crystalline drug was present in the LCT emulsion after digestion. However, in the case of the MCT emulsion, the situation was more complicated. In contrast to milk, infant formula, and LCT emulsion, after digestion of the MCT emulsion only two phases were observed, an aqueous supernatant layer and a pellet layer where excess drug and liquid crystalline phases resided (Figure S4).^14,25^ The implication of the sedimenting of the lipid to form a pellet rather than creaming to form a separate lipid layer means that it was not possible to discriminate between precipitated drug and drug solubilized in the lipid digestion phases. Therefore, the amount of drug in the ‘lipid+supernatant’ measurement is artefactually low for the MCT emulsion in Figure 3f as only drug solubilized in the aqueous layer could be independently determined, with the majority of digested lipids precipitating out with lopinavir in a dense pellet form.

### 3.3 Effects of Lopinavir on the Solubilization of Ritonavir in Lipid-Based Formulations during Digestion

**Figure 4:**
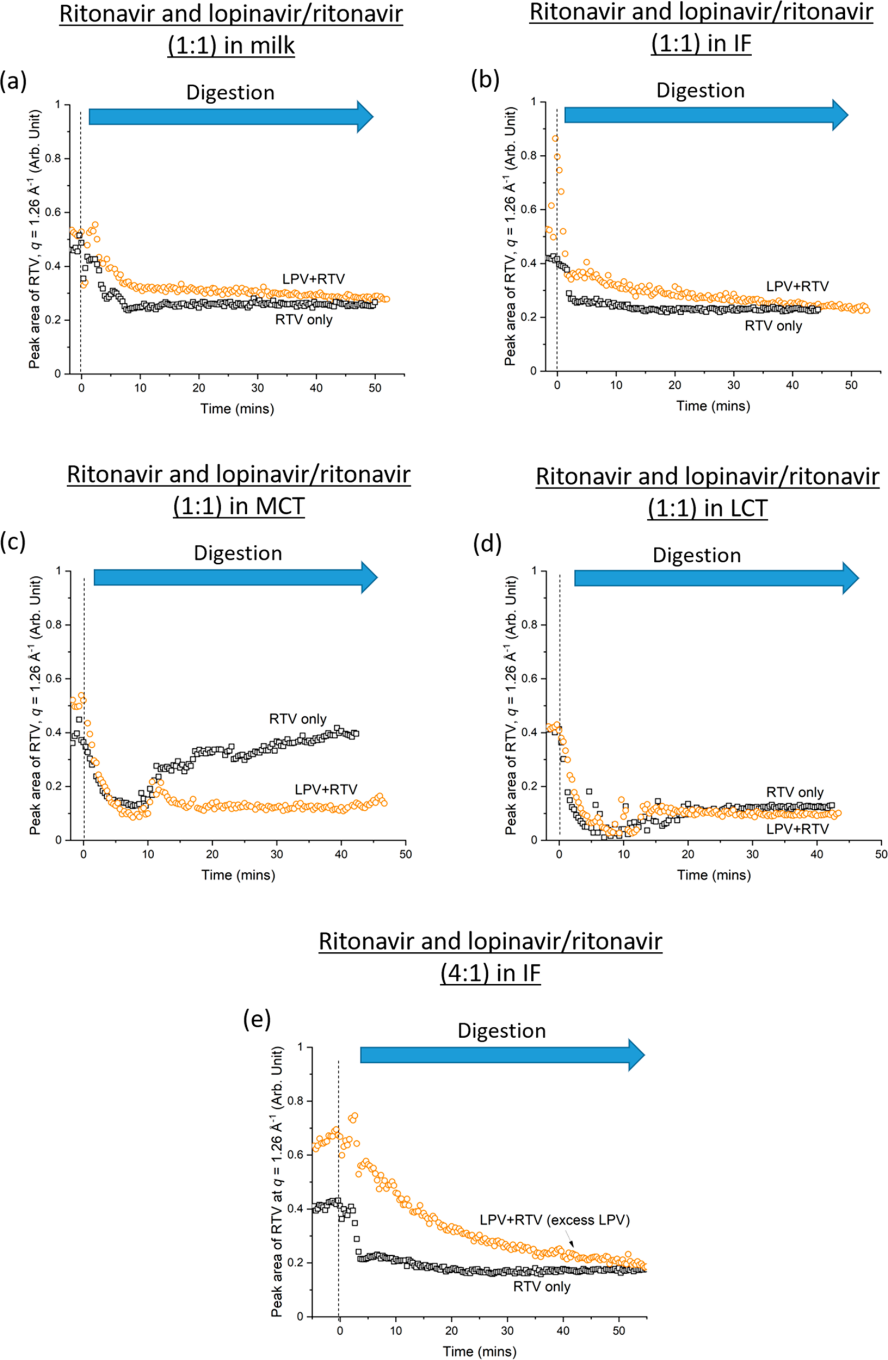
Residual crystalline ritonavir (determined by area under the diffraction peak of ritonavir at *q* = 1.26 Å^–1^) remaining during dispersion (time < 0 min) and digestion (time > 0 min) in the presence and absence of lopinavir in (a) milk (LPV:RTV = 1:1), (b) infant formula (IF; LPV:RTV = 1:1), (c) medium chain triglyceride emulsion (MCT; LPV:RTV = 1:1), and (d) long chain triglyceride emulsion (LCT; LPV:RTV = 1:1). (e) Effects of excess lopinavir (LPV:RTV = 4:1) on residual crystalline ritonavir during digestion of infant formula. Pancreatic lipase was injected at 0 min in all cases.

Figure 4 shows the solubilization behavior of ritonavir in the lipid-based formulations during digestion. In general, digestion of the lipid-based formulations (milk, infant formula, and LCT emulsion) resulted in a decrease in the characteristic peak area of ritonavir (*q* = 1.26 Å^–1^). The exception was the case of ritonavir+MCT where drug precipitation occurred (peak area increased during digestion). The results also indicated that the area of the characteristic ritonavir peak (i.e., the amount of crystalline ritonavir present) was generally larger when lopinavir was added to the system prior to lipase injection (time <0 min) but comparable values were observed after digestion. Similar trends were observed when excess lopinavir was present at 4:1 lopinavir:ritonavir weight ratios, as seen in Figure 4e. Hence, lopinavir reduced the solubilization of ritonavir during early digestion, but this effect was overcome as digestion progressed.

Solubilization of ritonavir was incomplete in all the lipid formulations tested, regardless of whether lopinavir was also present (Figure 4). While complete or near complete solubilization of lopinavir could be achieved in triglyceride emulsions containing 3.8 w/v% fat (Figure 3d and e), an increase in fat content from 3.8 w/v% (75.2 mg ritonavir/g lipid) to 6.3 w/v% (45.4 mg ritonavir/g lipid) still did not improve the solubilization of ritonavir (Figure S5).

**Figure 5:**
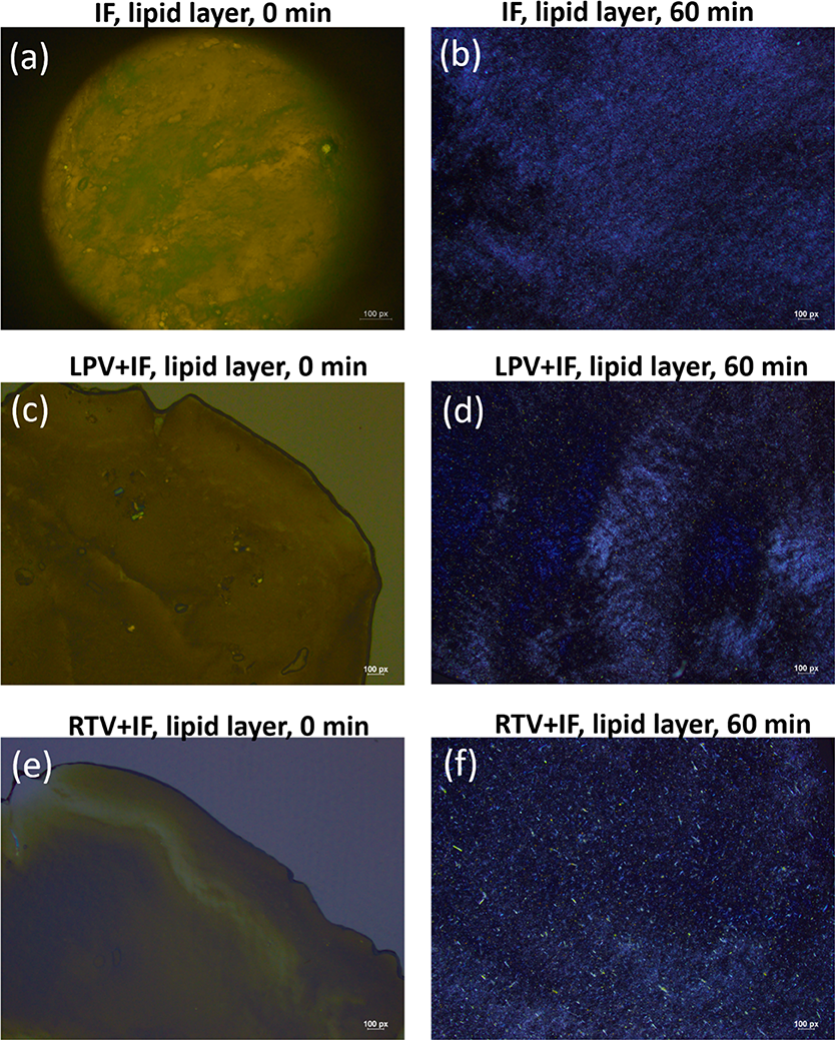
Polarized microscopy images of the separated lipid layers of infant formula before (0 min) and after (60 min) digestion for samples containing (a, b) no drugs, (c, d) lopinavir, and (e, f) ritonavir. Ritonavir needle-shaped crystals were partitioned in the digested lipid layer (panel f). Scale bar: 100 px = 120 μm.

Attempts to use HPLC to quantify the amount of ritonavir in the different layers of digested infant formula after centrifugation was complicated by the preferential localization of ritonavir crystals in the upper lipid phase after ultracentrifugation. Unlike lopinavir, where excess drug crystals sedimented into the bottom of the separated phases, ritonavir crystals were found to partially reside in the upper lipid phase after digestion. This is shown in the crossed polarized microscope images in Figure 5f of the lipid phase of ritonavir+infant formula after digestion, which clearly show the presence of ritonavir crystals. This effect appears to be limited to the postdigestion case and to ritonavir, as no drug crystals were seen in the lipid phase containing undigested triglycerides of the infant formula or for lopinavir before or after digestion (Figure 5e). This finding illustrates the difficulty in taking classical analytical “separate and sample” approaches for determining drug solubilization in these types of systems, which is not required for the *in situ* X-ray-based measurement.

## 4 Discussion

Oral bioavailability of active pharmaceutical ingredients is often limited by the poor solubility of the compounds in the gastrointestinal tract, thereby limiting the amount of drug available for absorption from the small intestine.^26^ Lopinavir and ritonavir are poorly water soluble protease inhibitors that have been used to treat HIV infection.^15,16^ Lopinavir is categorized either as a class 2 or class 4 drug in the Biopharmaceutics Classification System (BCS).^27^ Lopinavir has an aqueous solubility of about 3–4 μg/mL, which is not significantly affected by pH due to the nonionizable nature of the molecule.^28^ Meanwhile, ritonavir is a weakly basic drug with thiazole moieties that can be ionized at gastric pH (p*K*_a_ 1.8 and 2.6) but is un-ionized at the pH of the small intestine. Its aqueous solubility at pH 1.2 and 7.0 has been reported to be about 400 μg/mL and 2 μg/mL respectively,^28^ and is categorized as a BCS class 4 drug.^27^ The poorly water soluble nature of lopinavir and ritonavir has therefore led to the development of various formulation strategies to improve their oral bioavailability. These include, among others, amorphous solid dispersions^28,29^ and lipid-based formulations.^30,31^

Milk and infant formula-based formulations are gaining interest as a formulation option for poorly water soluble drugs for use in pediatric and low economy settings due to their favorable safety and cost attributes compared to commercial lipid formulations containing surfactants and solvents.^2^ With the renewed appreciation of the importance of digestion in understanding their performance as well as contemporary approaches for *in situ* measurement of drug solubilization during digestion, the development of these materials as drug delivery systems is gaining momentum. Notably, infant formula is manufactured in a highly regulated environment as a dry preparation, providing the opportunity for its use as an excipient for solid dosage forms, such as blended powder in a sachet or a dispersible tablet. It has a further advantage in providing potential for taste masking; ritonavir has been reported as having a bitter taste and is recommended to be given to children with chocolate milk.

Consequently, we investigated the solubilization behaviors of lopinavir and ritonavir in milk-based lipid formulations during digestion using *in situ* X-ray scattering. These studies are necessary to determine whether milk-based formulations are expected to provide a benefit in enhancing the solubilization of lopinavir/ritonavir in these complex media during and after digestion. It should be noted that while a broad correlation between drug solubility and oral bioavailability can generally be drawn (and that lopinavir and ritonavir are more soluble in the undigested milk-based formulations compared to aqueous solution; Figure S6), the data show that the drug is not completely dissolved in the formulations prior to digestion, so dissolution into the lipid phase prior to digestion is not anticipated to completely drive absorption and digestion is a critical requirement to provision of a solubilizing environment to maximize solubilization and consequently absorption. Solubilization of poorly water soluble drugs in these systems is dominated by the identity of the triglycerides present in the lipid droplets in the emulsions, specifically the monoglycerides and fatty acids produced on digestion.^32^ Thus, it was expected that different milk-like systems and lipid emulsions would have different effectiveness in solubilizing the two drugs during digestion. The studies presented here indicate that lopinavir is far more effectively solubilized by medium chain lipids (e.g., in the MCT emulsion). Our previous studies using MCT as a vehicle for artefenomel (performed under the same *in vitro* digestion conditions as used here) showed that artefenomel was more efficiently solubilized in the digested MCT than in LCT.^14^ This is consistent with the studies presented here and suggests that MCT fortified infant formula may present a particularly useful vehicle for the delivery of lopinavir/ritonavir combination therapy.

Generally, digestion of milk and infant formula with 3.8% (w/v) fat (75.2 mg of drug/g of fat) did not exert a significant impact on the solubilization of lopinavir, but addition of ritonavir to the sample mixtures improved the overall solubilization of lopinavir. This can be observed from the decrease in peak area characteristic of lopinavir, i.e., loss of drug crystallinity, after digestion of infant formula containing lopinavir/ritonavir mixed drugs at both 1:1 and 1:4 (excess ritonavir relative to lopinavir) weight ratios (Figure 3). Our findings suggest that increased plasma exposures of lopinavir following coadministration with ritonavir may not be caused solely by the inhibition of cytochrome P450 3A (CYP3A) enzyme-mediated metabolism of lopinavir but also the synergistic effect on drug solubilization. Interestingly, addition of lopinavir to ritonavir reduced the solubilization of ritonavir during early stages of digestion, but these effects were diminished as digestion progressed. It was therefore postulated that specific interactions between lopinavir, ritonavir, and milk digestion products exist although the mechanisms by which these interactions result in solubilization of lopinavir are unclear. Future molecular dynamic simulations could shed light on these complex interactions, for example the potential importance of hydrogen bonding partners, as lopinavir and ritonavir molecules exhibit multiple functional groups amenable to hydrogen bonding.^15,23^

Besides the impact of one drug on the other, the type and amount of lipids present also plays an important role in determining the extent of drug solubilization, which could point to the potential impact of food on the oral bioavailability of lopinavir and ritonavir. Results from our studies suggested that while both medium chain and long chain triglyceride emulsions enhanced the solubilization of lopinavir and ritonavir during digestion, the extent of drug solubilization was greater in lopinavir when compared to ritonavir in a mixed drug system (log *P*_lopinavir_ = 4.56 and log *P*_ritonavir_ = 5.98).^33^ These observations agree well with previous findings, where lopinavir was more soluble in fed-state human intestinal fluid compared to ritonavir.^34^ It was also shown that both lopinavir and ritonavir possess greater drug solubility in the fed-state human intestinal fluid compared to the fasted state, which was consistent with our findings, where lopinavir and ritonavir are poorly soluble in the blank samples when no lipids were present (Figure S7).

In particular, we demonstrated that digestion of LCT led to greater solubilization of ritonavir compared to MCT, which (as was expected) was further enhanced upon addition of bile salts due to micellar solubilization into the long chain fatty acids/bile salts mixed micelles (Figure 4 and S5). Additionally, our studies showed that in the absence of lopinavir precipitation of ritonavir occurred during digestion in MCT emulsions (Figure 4c). This finding is interesting in light of the potential relationship between drug precipitation and the lamellar phase formed during digestion of MCT, where integration of the lamellar peak formed at *q* = 0.21 Å^–1^ (Figure S8) showed that an increase in the lamellar peak area due to the formation of fatty acid-calcium soaps coincided with the decrease of the ritonavir peak area. This observation hinted at an interaction of ritonavir crystals with the lamellar structure, resulting in fewer drug crystals in suspension being exposed to the X-ray beam. Preferential localization of ritonavir (but not lopinavir despite the similar densities) in the lamellar phase soaps was observed from the microscopy images in Figure 5. However, as the digestion progressed, the intensity of the lamellar peak decreased and vesicular colloidal structures were formed presumably at the expense of calcium soaps, which was suggested to trigger the release of the ritonavir crystals.

Finally, it should be noted that although our findings showed the impact of digestion of lipids on solubilization of lopinavir and ritonavir in mixed drug settings (which were consistent with other BCS class 2 and class 4 drugs) that may lead to improved oral bioavailability, there is a gap in the literature directly relating the postdigestion solubilization afforded by milk-like systems with absorption which would need to still be clearly established as the literature is conflicting. It is however understood with confidence that a failure to provide drug in a solubilized form for such poorly soluble drugs will certainly be limiting for bioavailability. For example, administration of lopinavir/ritonavir with a moderate fat meal increased the exposure (AUC) of lopinavir by about 60% relative to fasting conditions when formulated as a soft gelatin capsule, compared to about 27% for the lopinavir/ritonavir tablet. A similar effect was observed for ritonavir with an increased AUC of about 15–24% following administration of the drug tablet.^35^ In another study, a reduction in the exposure of ritonavir was observed when a fatty meal was administered with the ritonavir powder formulation.^36^ Therefore, effects of formulation excipients on drug solubilization should also be systematically assessed. Nevertheless, our studies confirmed that solubilization of lopinavir/ritonavir in lipid-based formulations was affected not only by the amount and types of lipid in the formulations but also the presence of partner drug and the relative ratios of lopinavir to ritonavir in the mixed drug system.

## 5 Conclusions

In this study, the effects of ritonavir on the solubilization of lopinavir (and vice versa) during digestion of milk and infant formula, as prototypical pediatric friendly lipid formulations, were determined. Lipids enhanced the *in vitro* solubilization of lopinavir and ritonavir in a mixed drug setting during digestion and the solubilization of lopinavir and ritonavir were influenced by the presence of one another. Specifically, the solubilization of lopinavir was enhanced during the digestion of formulations containing ritonavir, suggesting the potential for solubilization to add to the enzymatic inhibition effect of ritonavir that is an accepted mechanism for boosting the bioavailability of lopinavir. The studies also showed that the digestion of MCT enhanced the solubilization of lopinavir, while for ritonavir, LCT was favored over MCT from a solubilization standpoint. The studies present further weight to the potential for milk-lipid-based formulations to deliver lopinavir and ritonavir in combination to children.

## Supporting Information Available

Micrographs of crystalline reference materials; X-ray diffraction data; time-resolved diffraction data showing absence of polymorphic transformations during digestion or exposure to gastric conditions; dependence of kinetics of drug dissolution on MCT content; photographs of ultracentrifuged formulations showing presence/absence of pellet phase; dependence of kinetics of drug dissolution on type of triglyceride present; partitioning of drugs in undigested milk and infant formula; kinetics of drug recrystallization during digestion; link between ritonavir solubilization and lamellar phase (PDF)

## Abbreviations

SAXS: small-angle X-ray scattering
LPV: lopinavir
RTV: ritonavir
IF: infant formula
MCT: medium chain triglycerides
LCT: long chain triglycerides
NaTDC: sodium taurodeoxycholate
DOPC: 1,2-dioleoyl-*sn*-glycero-3-phosphocholine
