# Supplementary material for: Implications of the Digestion of Milk-Based Formulations for the Solubilization of Lopinavir/Ritonavir in a Combination Therapy
Source: Mol Pharm. 2023 Mar 15;20(4):2256–65. doi: 10.1021/acs.molpharmaceut.3c00072 (PMC10074382; doi:10.1021/acs.molpharmaceut.3c00072)
Supplement: Supplementary file 1 — mp3c00072_si_001.pdf [file mp3c00072_si_001.pdf]

Supporting Information

Implications of the digestion of milk-based formulations for the solubilisation of lopinavir/ritonavir in a combination therapy

Malinda Salim,[1] Gisela Ramirez,[1] Andrew J. Clulow,[1,2] Adrian Hawley,[2] and Ben J. Boyd[1,3*]

[1]Drug Delivery, Disposition and Dynamics, Monash Institute of Pharmaceutical Sciences, Monash, 381 Royal Parade, Parkville, VIC 3052, Australia

[2]Australian Synchrotron, ANSTO, 800 Blackburn Rd, Clayton, VIC 3168, Australia

[3]Department of Pharmacy, University of Copenhagen, Universitetsparken 2, 2100 Copenhagen, Denmark

*Corresponding author details:

Postal Address: Monash Institute of Pharmaceutical Sciences, Monash University (Parkville Campus), 381 Royal Parade, Parkville, VIC 3052, Australia

Telephone: +61 3 99039112; Fax: +61 3 99039583

Email: ben.boyd@monash.edu

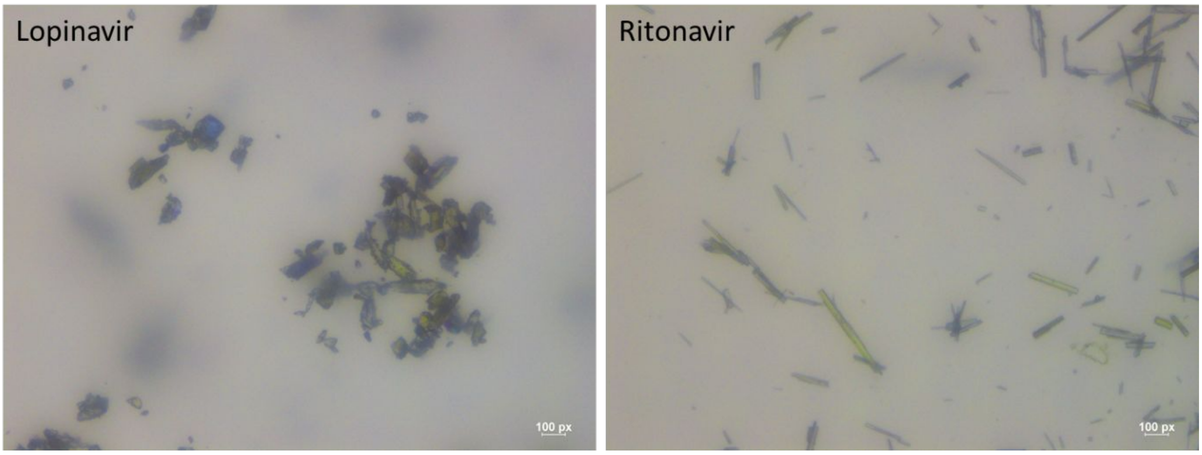

Figure S1. Microscopic images of non-micronised lopinavir and ritonavir powders. Scale bar: 100 px = 25 μm

Table S1. X-ray scattering peak positions of lopinavir and ritonavir powders

Lopinavir q (Å^{-1})	Ritonavir q (Å^{-1})
0.35	0.46
0.46	0.61
0.53	0.67
0.63	0.70
0.70	0.78
0.88	0.92
0.92	0.94
1.06	0.97
1.11	0.98
1.18	1.06
1.26	1.11
1.29	1.14
1.33	1.16
1.37	1.18
1.40	1.23
1.43	1.26
1.47	1.30
1.49	1.34
1.56	1.38
1.58	1.40
1.62	1.42
1.65	1.44
1.70	1.46
1.71	1.48
1.73	1.50
1.74	1.52
1.82	1.53
1.83	1.57
1.86	1.59
1.90	1.61
	1.63
	1.66
	1.67
	1.70
	1.71
	1.73
	1.75
	1.75
	1.79
	1.82
	1.84

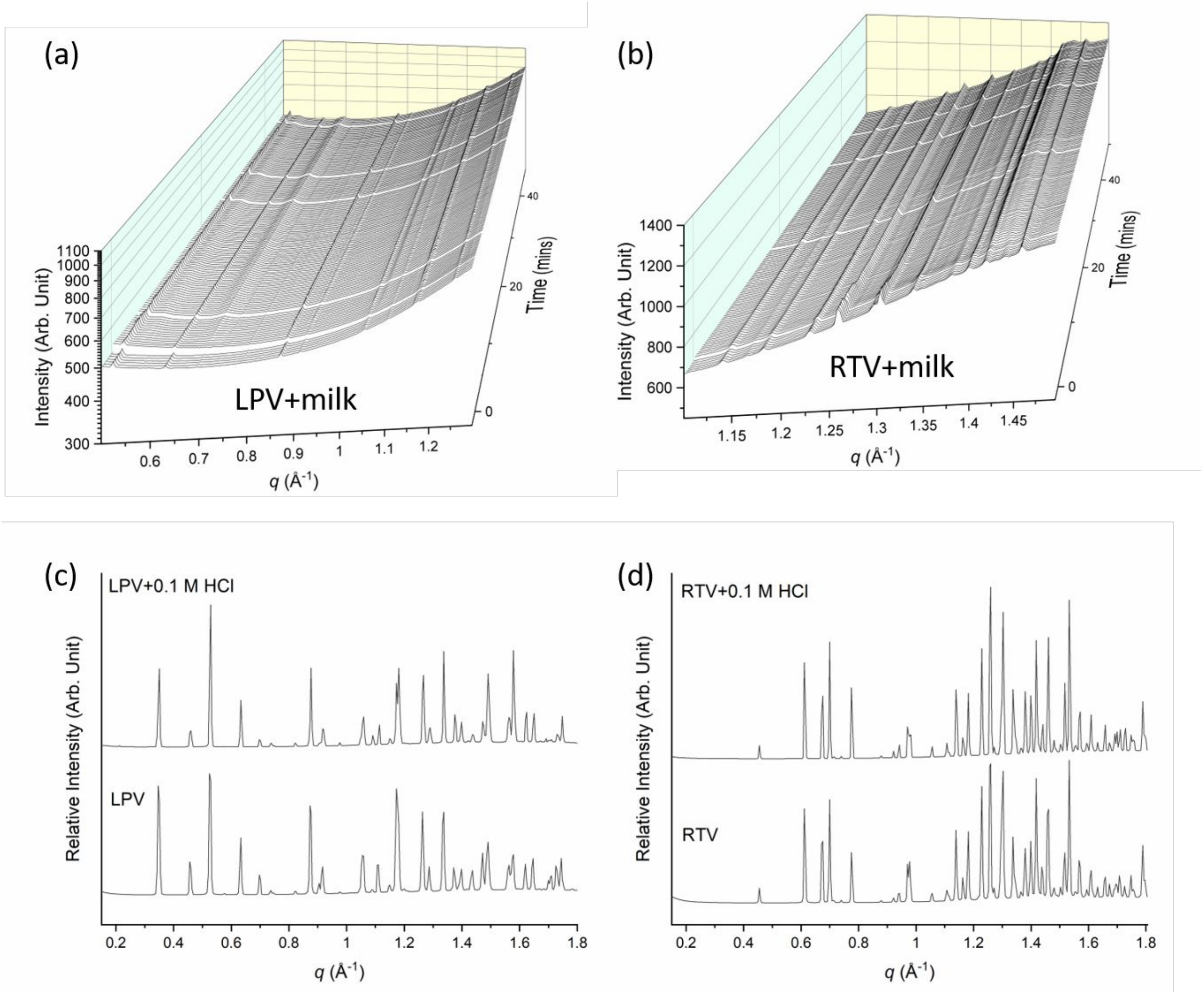

Figure S2. Examples of the X-ray scattering patterns for (a) lopinavir (LPV) and (b) ritonavir (RTV) during digestion in milk. No disappearance of the initial Bragg peaks and appearance of new Bragg peaks at different *q* were observed during the course of digestion, indicating no polymorphic transformations. Changes in the peak positions for (c) lopinavir and (d) ritonavir after dispersions in 0.1 M HCl were also not observed.

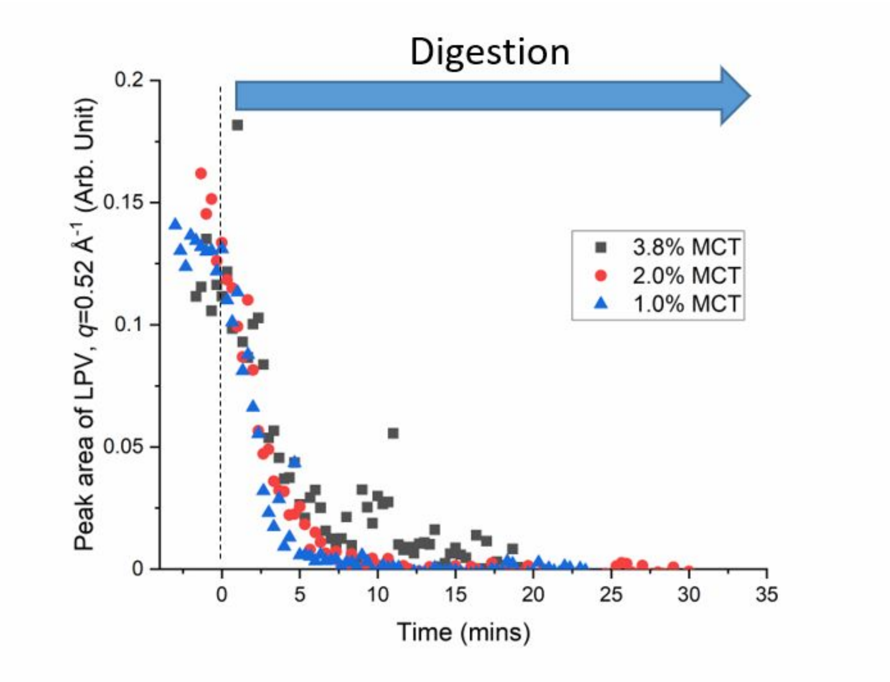

Figure S3. Residual crystalline lopinavir remaining during dispersion (time < 0 min) and digestion (time > 0 min) of medium chain triglycerides (MCT) with different fat content: 3.8% (75.2 mg drug/g fat), 2.0% (142.9 mg drug/g fat), and 1.0% (285.7 mg drug/g fat). Pancreatic lipase was injected at 0 min.

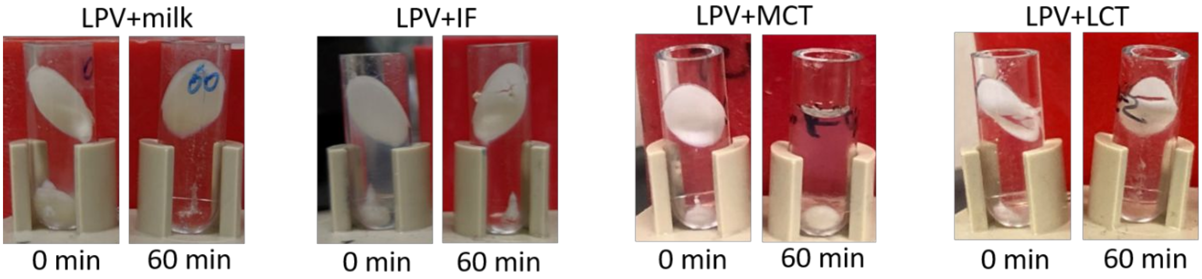

Figure S4. Phase separation of undigested (0 min) and digested (60 min) milk, infant formula (IF), medium chain triglycerides (MCT) and long chain triglycerides (LCT) emulsions using ultracentrifugation. Upper lipid layer, middle aqueous supernatant layer, and bottom pellet layer was observed for all the lipid-based formulations before and after digestion except for the 60 min digested MCT where no upper lipid layer was present.

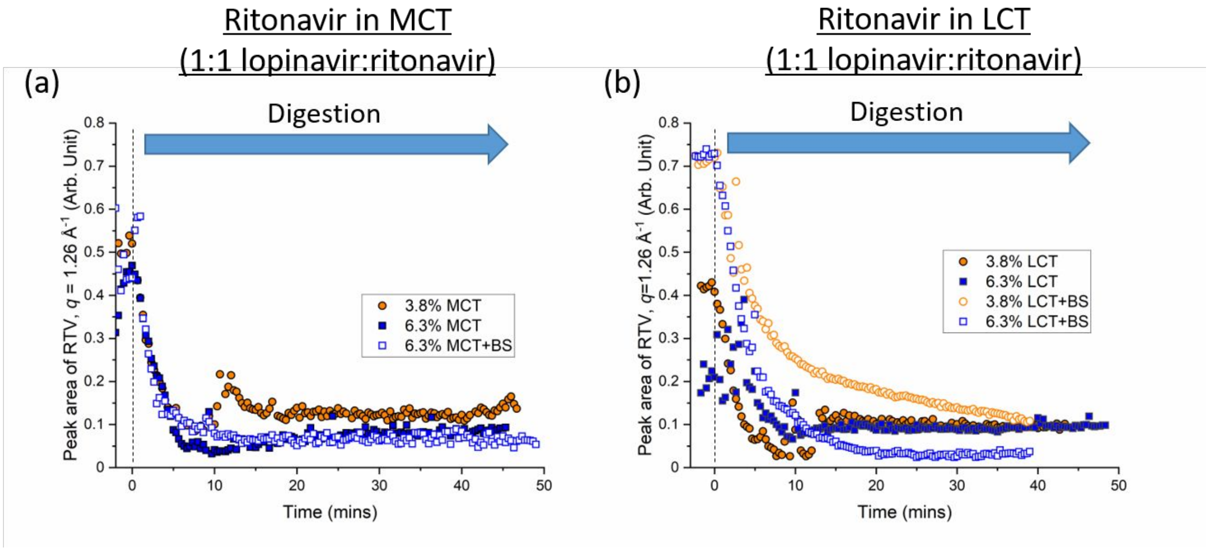

Figure S5. Residual crystalline ritonavir remaining during dispersion (time < 0 min) and digestion (time > 0 min) of (a) medium chain triglycerides (MCT) and (b) long chain triglycerides (LCT) at 3.8% (75.2 mg drug/g fat) and 6.3% (45.4 mg drug/g fat) fat in presence and absence of 4.7 mM NaTDC/0.98 mM DOPC bile salt micelles (BS). Pancreatic lipase was injected at 0 min.

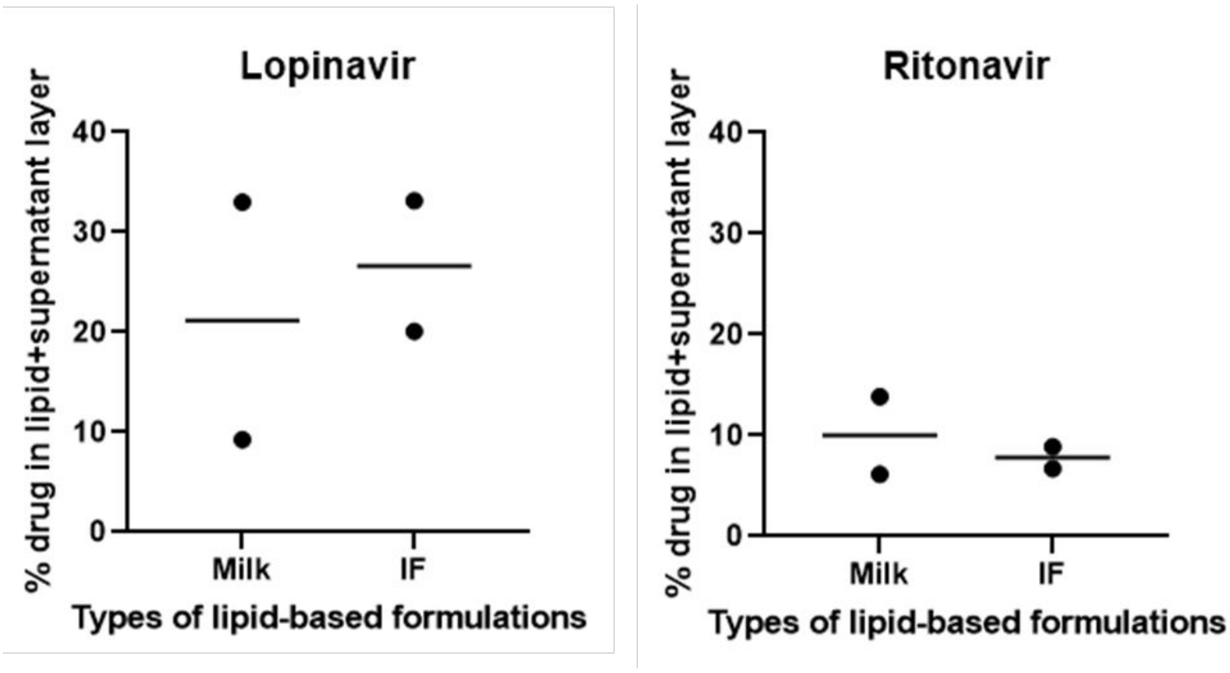

Figure S6. Partitioning of lopinavir and ritonavir in lipid+supernatant layers of the undigested milk and infant formula at 0 min prior to the injection of pancreatic lipase (average based on n=2).

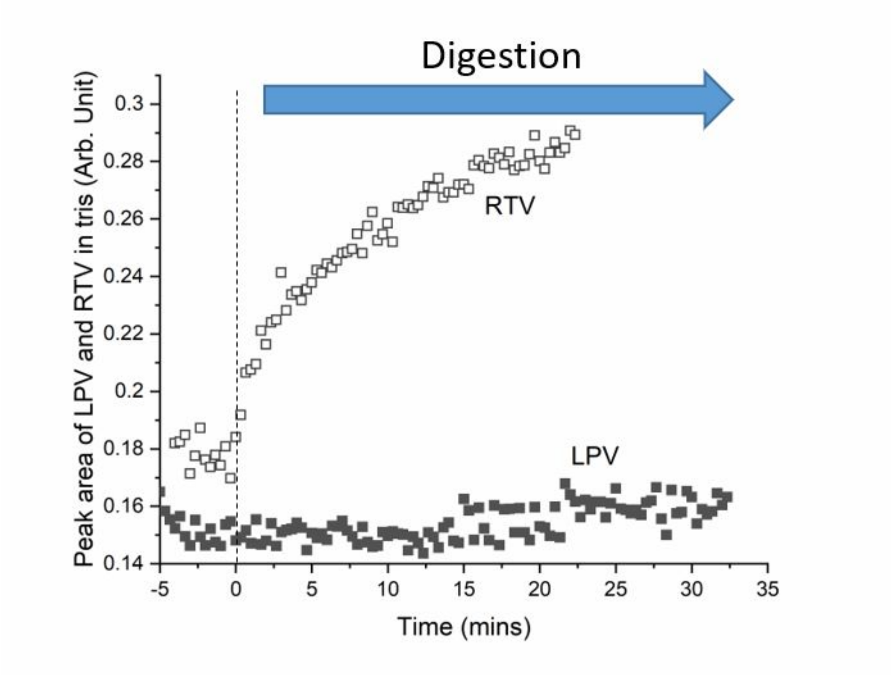

Figure S7. Residual crystalline lopinavir (LPV; peak area at $q = 0.52$ Å^{-1}) and ritonavir (RTV; peak area at $q = 1.26$ Å^{-1}) during dispersion (time < 0 min) and digestion (time > 0 min) in tris buffer containing no lipid. Pancreatic lipase was injected at 0 min. An increase in peak area of ritonavir during digestion suggested that re-precipitation of ritonavir in the intestinal pH may not have reached equilibrium prior to the lipase injection.

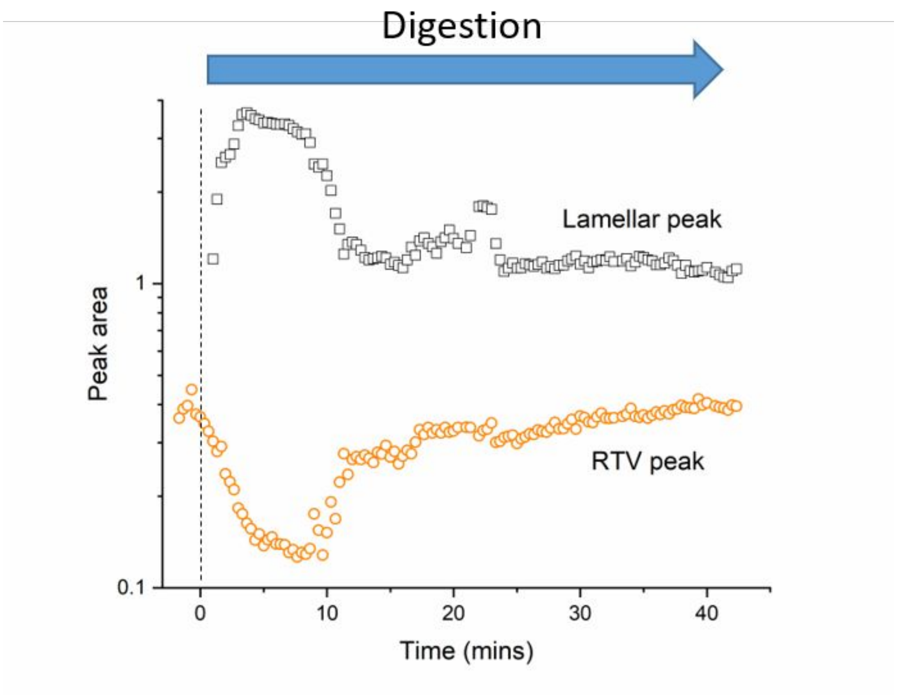

Figure S8. Changes in the peak area of ritonavir (RTV; $q = 1.26$ Å^{-1}) in relation to the liquid crystalline lamellar phase (lamellar peak at $q = 0.21$ Å^{-1}) during digestion in medium chain triglycerides (MCT). Pancreatic lipase was injection at 0 min.
